# Gut Microbiota and Enteral Nutrition Tolerance in Non-Abdominal Infection Septic ICU Patients: An Observational Study

**DOI:** 10.3390/nu14245342

**Published:** 2022-12-16

**Authors:** Wen Xu, Ming Zhong, Tingting Pan, Hongping Qu, Erzhen Chen

**Affiliations:** 1 Department of Critical Care Medicine, Ruijin Hospital, Shanghai Jiao Tong University School of Medicine, Shanghai 200020, China; 2 Department of Emergency, Ruijin Hospital, Shanghai Jiao Tong University School of Medicine, Shanghai 200020, China

**Keywords:** enteral nutrition tolerance, sepsis, microbiota

## Abstract

**Background:** The effect of gut microbiota on enteral nutrition tolerance in critically ill patients is unclear. **Methods:** Non-abdominal sepsis patients in an ICU, sorted by whether they reached 20 Kcal/kg/day on the 3rd day of EN, were divided into tolerance and intolerance groups. Their feces on day 1 and day 3 of EN initiation were collected for 16s rDNA and short-chain fatty acid (SCFA) testing. **Results:** There were 14 patients included in the tolerance group and 10 in the intolerance group. On EN day 1, the OTUs and microbiota diversity were higher in the tolerance group than in the intolerance group. The ratio of Firmicutes to Bacteroidetes was higher in the intolerance group on EN day 1. The genus Parabacteroides were the most significantly elevated in the tolerance group. On EN day 3, the genus Escherichia-Shigella was the most significantly elevated in the tolerance group. On EN day 3, the levels of SCFA decreased more significantly in the intolerance group. **Conclusion:** Enteral nutrition tolerance is associated with microbiota features and short-chain fatty acid levels. A higher ratio of Firmicutes to Bacteroidetes and microbiota diversity on EN day 1 may help in the early prediction of EN tolerance.

## 1. Introduction

Enteral nutrition (EN) is a fundamental support therapy in critically ill patients. Enteral nutrition can help to reduce in-hospital mortality and acquired infection in critical illnesses [1]. “If the gut can safely work, use it” is regarded as a golden rule of nutrition support [2]. Several challenges need to be overcome to apply EN effectively. Feeding intolerance is one of the most important challenges in severely ill patients [3]. The incidence of enteral nutrition intolerance is about 40%, and its occurrence is related to poor outcomes and prolonged hospitalization [3,4,5] in critically ill patients. Intestinal flora is an important structure for the function of the gastrointestinal tract, which is involved in regulating intestinal motility, digestion, immunity, and so on [6]. It is thought that gut microbiota regulates the human intestinal tract and the whole body through its metabolites, in which short-chain fatty acids (SCFAs) are closely related to intestinal motility [7]. The current evidence about EN tolerance has a lack of data on gut microbiota [4,5]. The relationship between gut tolerance and intestinal flora and metabolites in critically ill patients remains unclear. Meanwhile, products such as probiotics and prebiotics achieve much attention in the clinic, but the mechanism and how to use them properly are important topics [7]. The first step should be figuring out the microbiota characteristics in both EN tolerant and intolerant patients. We hypothesize that gut microbiota characteristics are different between enteral tolerant and intolerant patients in the early stage of enteral nutrition; thus, we conducted this investigation to prove the assumptions.

## 2. Method

This study was a single-center observational study, recruiting patients from June 2019 to June 2020. On the day of EN (day 1) and 3 days after the beginning of EN (day 3), the fecal samples were collected for 16s rDNA test, and the fecal short-chain fatty acids were tested on day 3. Based on whether they reached 20 Kcal/kg/day on day 3, patients were divided into tolerance and intolerance groups, and the profiles of their gut microbiota composition and SCFA content were compared. Both groups were treated according to the clinical standard treatment procedure without additional intervention.

The standard treatment in our ICU includes a proton pump inhibitor, prokinetic agents (Trimebutine Maleate 100 mg tid), nasogastric tube feeding initially, and polymetric EN formula with fiber and whole proteins. A patient was anticipated to achieve 20 Kcal/kg/day on Day 3 if he or she was without any contraindication judged by the ICU doctor in charge. AGI assessment was made by doctors. Maximal AGI degree over the 3 days was used to describe the clinical GI function.

### 2.1. Ethics Approval

The study protocol was approved by the Ruijin Hospital Ethics Committee of Shanghai Jiaotong University School of Medicine, China. Formal informed consent was obtained from the patients or their next of kin. Data can be obtained by contacting the authors.

### 2.2. Inclusion Criteria

(1). Admission to ICU for sepsis. Sepsis was defined according to the 2016 Sepsis 3.0 definition.

(2). Undergoing enteral nutrition support. 

(3). Age 30~70 years.

### 2.3. Exclusion Criteria

(1). A sepsis caused by intra-abdominal infections including the following: intestinal obstruction, gastrointestinal perforation, necrotizing enteritis, toxic megacolon with infection, intestinal fistula, and clostridium difficile-associated enteritis.

(2). Other history involving gastrointestinal disease: active gastrointestinal bleeding, intestinal necrosis, ulcerative colitis, radioactive enteritis, inflammatory bowel disease, short bowel, and gastrointestinal surgery within half a year.

(3). Untreated treatment malignancies, autoimmune diseases, AIDS.

(4). Pregnancy.

(5). Patients with less than 72 h of enteral nutrition due to any reason. 

### 2.4. 16s rDNA Analyses

We used the patients’ feces as samples for 16s rDNA testing. The feces were collected as soon as possible after patient defecation. The samples were put in a refrigerator at −80° for preservation and were tested within 2 weeks.

#### 2.4.1. Stool Sample Processing and DNA Extraction

Total genome DNA from samples was extracted using the CTAB/SDS method. DNA concentration and purity were monitored on 1% agarose gels. According to the concentration, DNA was diluted to 1 ng/μL using sterile water.

#### 2.4.2. Amplicon Generation

16s rDNA genes were amplified using the specific primer with the barcode. All PCR reactions were carried out in 30 μL reactions with 15 μL of Phusion (High-Fidelity PCR Master Mix, New England Biolabs).

#### 2.4.3. Sequencing

Sequencing libraries were generated using NEB Next Ultra DNA Library Prep Kit for Illumina (NEB, USA) following the manufacturer’s recommendations. The library was sequenced on an Illumina Miseq/HiSeq2500 platform, and 250 bp/300 bp paired-end reads were generated. 

### 2.5. SFCA Measurement

The levels of SFCAs were measured using gas chromatography–mass spectrometry (GC-MS) by single ion monitoring. The samples were analyzed with a GC-MS detector (7890A/5975B; Agilent, Santa Clara, CA, USA) and an HP-FFAP capillary column (DB-WAX 30 m × 0.25 mm ID × 0.25 um; Agilent, Santa Clara, CA, USA)

### 2.6. Statistical Strategy

For 16s rDNA results, sequence analyses were performed by the UPARSE software package using the UPARSE-OTU and UPARSE-OTUref algorithms. In-house Perl scripts were used to analyze alpha and beta diversity. We analyzed the phylum and genus levels. Comparative analyses of the alpha diversity index (Shannon index, Simpson index) were performed using Student’s *t*-test. Interindividual variability (beta diversity) at the OTU level among groups was evaluated by weighted unifrac distance using QIIME. Principal coordinate analysis (PCoA) was based on weighted unifrac distance. The statistical analysis of taxonomic and functional profiles (STAMP) analysis method was used to determine statistically different bacteria among groups [8]. 

SPSS 22.0 (SPSS Inc., Chicago, IL, USA) software was used for the clinical data and SCFA analysis. Data were presented as median (interquartile range (IQR)), as they had a non-Gaussian distribution. As for comparisons between groups, Mann–Whitney U test was used for continuous variables, and the chi-square test was performed for categorical variables.

## 3. Result

### 3.1. Clinical Characteristics

A total of 24 patients were admitted. The median age of the patients was 62 (51–70). The number of patients who reached enteral nutrition tolerance was 14, and 10 patients did not reach enteral nutrition tolerance. There were four deaths in the tolerance group and four deaths in the intolerance group (Table 1).

### 3.2. Gut Microbiota Composition on Day 1 and Day 3

#### 3.2.1. Relative Abundance of Patients on Phylum and Genus Level

The top ten relative abundances of the tolerant and the intolerant patients on the phylum level and genus level are listed in Table 2. 

**Figure 1:**
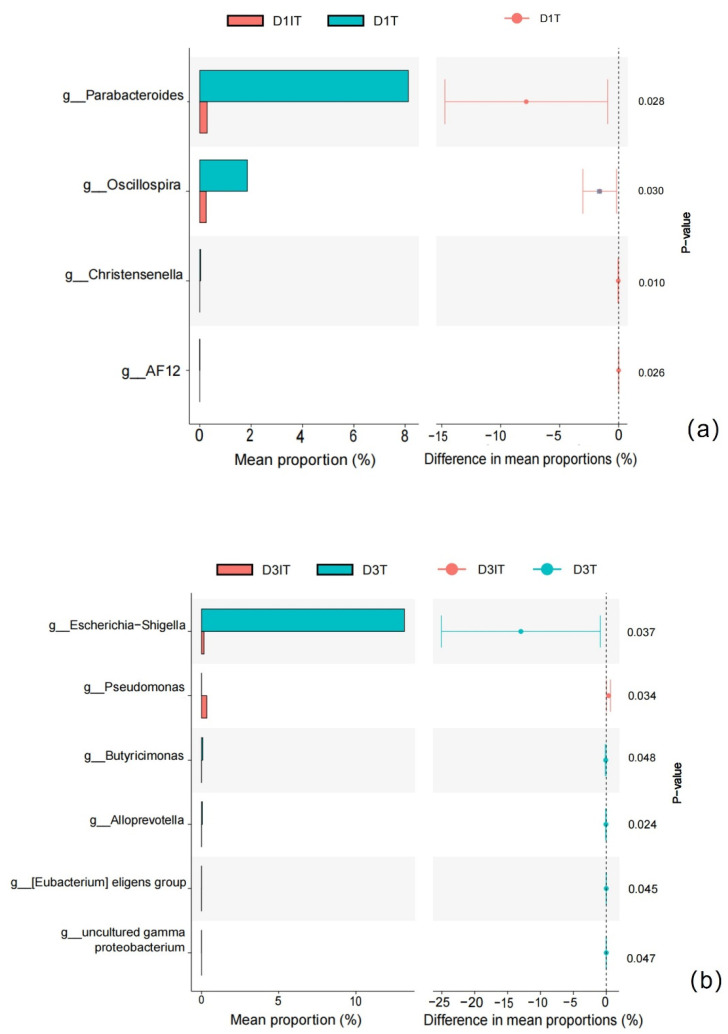
(**a**) Major differential bacterial genera between tolerant and intolerant patients on day 1 by STAMP. D1T: day 1 tolerance group; D1IT: day 1 intolerance group. (**b**) Major differential bacterial genera between tolerant and intolerant patients on day 3 by STAMP. D3T: day 3 tolerance group; D3IT: day 3 intolerance group.

On day 1, phylum Bacteroidetes was higher in the tolerance group. Genus Enterococcus and genus Klebsiella were lower in the tolerance group; genus Parabacteroides and genus Bacteroides were higher in the tolerance group. Genus Parabacteroides was indicated as the major differential genus by STAMP analysis (Figure 1a).

On day 3, phylum Bacteroidetes, phylum Verrucomicrobia, and phylum Actinobacteria were higher in the tolerance group. Genus Enterococcus, genus Sphingomonas, and genus Klebsiella were lower in the tolerance group; genus Escherichia-Shigella and genus Bifidobacterium were higher in the tolerance group. Genus Escherichia-Shigella and genus Pseudomonas were the major differential genera in STAMP analysis (Figure 1b).

#### 3.2.2. Firmicutes/Bacteroidetes Ratio (F/B) in Tolerance Group and Intolerance Group

Firmicutes/Bacteroidetes ratio (F/B) was used to describe the degree of alteration of the gut microbiota. The F/B was calculated by the relative abundance of phylum Firmicutes divided by the relative abundance of phylum Bacteroidetes. The F/B was higher in the intolerance group on day 1 and was similar in both groups on day 3 (Table 3). 

### 3.3. Gut Microbiota Diversity on Day 1 and Day 3

There were 2038 OTUs in the tolerance group and 1795 OTUs in the intolerance group on day 1. On day 3, there were 1434 OTUs in the tolerance and 1113 OTUs in the intolerance group.

#### 3.3.1. Alpha Diversity

The alpha diversity of the tolerance group on day 1 was higher than that of the intolerance group (Shannon index: tolerance group vs. intolerance group 3.86 (2.75, 4.81) vs. 2.32 (1.79, 3.76), *p* = 0.03; Simpson index: tolerance group vs. intolerance group: 0.87 (0.70, 0.91) vs. 0.72 (0.53, 0.87), *p* = 0.12), while on day 3 there was no significant difference (Table 2, Shannon index, tolerance group vs. intolerance group: 2.95 (1.62, 3.84) vs. 2.55 (1.54, 3.33), *p* = 0.15; Simpson index, tolerance group vs. intolerance group: 0.66 (0.28, 0.80) vs. 0.71 (0.32, 0.81), *p* = 0.28). 

#### 3.3.2. Beta Diversity

**Figure 2:**
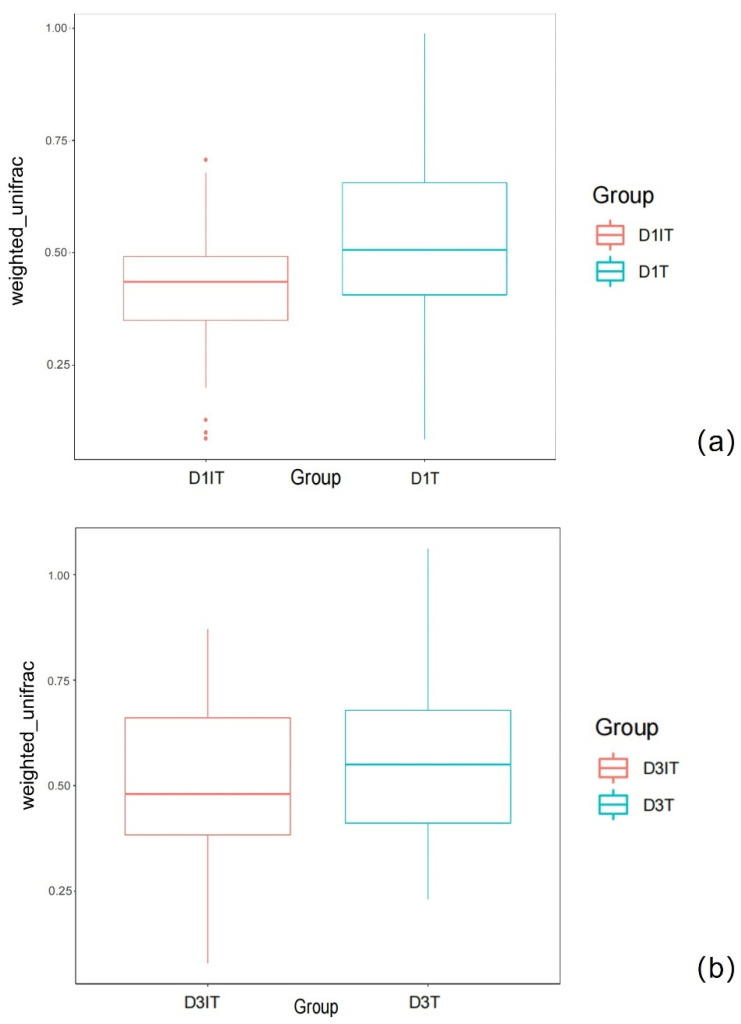
(**a**) The weighted unifrac comparison between tolerant and intolerant patients on day 1. D1T: tolerance group on day 1, D1IT: intolerance group on day 1. D1T vs. D1IT: 0.48 (0.05, 0.98) vs. 0.4 (0.20, 0.71), *p* < 0.01; (**b**) The weighted unifrac comparison between tolerant and intolerant patients on day 3. D3T: tolerance group on day 3, D1IT: intolerance group on day 3. D3T vs. D3IT: 0.51 (0.19, 1.03) vs. 0.48 (0.12, 0.82), *p* > 0.05.

The beta diversity was described by weighted unifrac analysis, which showed a significant difference between the two groups on day 1 (tolerance group vs. intolerance group, 0.48 (0.05, 0.98) vs. 0.4 (0.20, 0.71), *p* < 0.01, Figure 2a). On day 3, there was no significant difference (tolerance group vs. intolerance group, 0.51 (0.19, 1.03) vs. 0.48 (0.12, 0.82), *p* > 0.05, Figure 2b).

### 3.4. The Contents of Short-Chain Fatty Acids

The total amount of short-chain fatty acids in the tolerance group was significantly higher than that in the intolerance group on day 3 (Jonckheere–Terpstra test, Table 4). All kinds of short-chain fatty acids were generally more abundant in the tolerant group. Differences in propionic acid, isobutyric acid, isovaleric acid, butyric acid, and valeric acid were statistically significant between the two groups. The total SCFA content was negatively correlated with the F/B on day 1 (Spearman r = −0.58, *p* = 0.008).

## 4. Discussion

Enteral nutrition intolerance is one of the major challenges in the ICU. Studies have shown that enteral nutrition intolerance is associated with higher mortality, nosocomial infection rates, and longer ICU hospital stay [1]. The gut microbiota is a composition of intestinal functions with great importance. However, the relationship between gut microbiota and gut function is not clear in the critically ill population. Enteral nutrition tolerance is an important aspect in the implementation of gastrointestinal (GI) function. The gut microbiota had been studied profoundly in neonatal feeding intolerance, and great advancements had made in understanding and probiotic treatment [9]. Gut microbiota changes greatly with growth, from birth to adulthood [6]. Few data shed light on the characteristics of the gut microbiota in enteral nutrition intolerance in the ICU population. Therefore, it is crucial to know the features of this population in order to develop proper treatment. We designed this study to reflect the relationship between intestinal microflora and enteral nutrition tolerance in critically ill patients so that evidence can be given for future understanding and treatment targeting the gut microbiota in the adult ICU population.

In clinical practice, enteral nutrition intolerance can be judged by the symptoms or whether enteral nutrition reaches the target amount. According to the definition by ESICM [10], if a patient cannot be fed the target amount (20 Kcal/kg/day) in 72 h by enteral nutrition, the patient is diagnosed with enteral nutrition intolerance. We chose this definition in our ICU because the definition varied greatly among different studies using symptoms to diagnose EN intolerance [11], which may lead to bias among physicians. Several clinical features may be associated with EN intolerance. Heyland et al. [5] reported in an international survey that age, region (Asia), burns, and a high APACHE II score may be associated with EN intolerance. Hu et al. [12] reported 15 factors that may affect EN tolerance, such as pneumonia, shock, infection site, and EN formula. However, this study design was not suitable for studying the relationship between gut microbiota and EN tolerance, as the population had great heterogeneity, which may lead to difficulties in the analysis of the data. In this study, the actual amount of enteral nutrition in the two groups was significantly different, while the clinical characteristics such as nutrition risk, organ support, AGI level, SOFA, CRP, PCT, and infectious pathogen were similar. Although there was no EN protocol adopted in our ICU, our group shared common ideas and treatments in ICU nutrition, such as PPI usage, antibiotic usage, and initial EN formula. The risk of a systematic difference between groups was low, as was the heterogeneity of patients. Therefore, the results can reflect the relationship between enteral nutrition tolerance and the gut microbiota. 

As for the composition of gut microbiota, it showed differences between the two groups. On the phylum level, phylum Bacteroidetes had higher abundance in the tolerance group on both day 1 and day 3. Bacteroidetes and Firmicutes were reported as the most dominant members in the bacterial community of human gut microbiota on the phylum level; the Firmicutes/Bacteroidetes ratio (F/B) can be used to measure the degree of disturbance of the microbiota and is much easier for doctors to interpret than the whole landscape of the microbiome [13]. Oijma et al. [14] found that an F/B of either >10 or <0.1 is associated with death in critically ill patients, which means that a great disturbance in the microbiota may be associated with poor outcomes. In some studies, a lower F/B is associated with a lower level of inflammation [15]. In our study, we mainly used the third/fourth-generation cephalosporins and carbapenems as antibiotic regimes, which mainly affect Gram-negative bacteria (namely Bacteroidetes). This resulted in a lower F/B on day 1 than on day 3. We also found that F/B was higher in the intolerance group on day 1, while on day 3 the F/B values were similar in both groups. This indicated that F/B can be a predictor of enteral nutrition tolerance. However, more samples are needed to set up a cutoff value, which is necessary for further studies. 

On the genus level, genus Parabecteroid and genus Bacteroidetes had higher abundance in the tolerance group on day 1. Genus Parabecteroid was the major differential genus on day 1. This finding is consistent with the microbiota features on the phylum level. Genus Parabecteroid was recently recognized as a probiotic which is beneficial in metabolic diseases [14]. Our finding suggests it is worthwhile to further study if the genus Parabecteroid is good for EN tolerance in the ICU population. On Day 3, genus Escherichia-Shigella had a higher abundance in the tolerance group. This finding needs further confirmation, as higher Escherichia-Shigella abundance is usually associated with poorer gastrointestinal function. In addition, as most of our patients were treated with a proton pump inhibitor and broad-spectrum antibiotics, an increase in Escherichia-Shigella abundance is common in this situation. Genus Pseudomonas was more abundant in the intolerance group on day 3. Genus Pseudomonas is associated with gastrointestinal function impairment, which may result in EN tolerance.

The diversity of gut microbiota is an important reference for GI function, and it is generally considered that healthy patients have greater diversity. In the disease state, a decreased diversity of flora is often an early indicator of damage [16]. Alpha diversity evaluates the diversity within a sample, including richness and evenness measurements, and can be described by OTUs or Shannon Index [17]. Beta diversity evaluates differences in the microbiome among samples; weighted unifrac was used to describe it in this study. [18] Our results showed that there was a significant difference in alpha diversity (Shannon index) and beta diversity (weighted unifrac) between groups on day 1. On day 3, the diversity levels of the two groups were similar. This showed that the tolerant patients have better diversity, which is consistent with the reports for chronic diseases, suggesting a better state of GI function [19]. It also indicated that diversity on day 1 might be an early predictor for EN tolerance, but more data were needed to further prove this. 

It is now believed that the abnormal flora ultimately works through the metabolites of the flora. Short-chain fatty acids (SCFAs) are important substances that can be produced and used by flora [20] Our results showed that the total content of SCFAs in tolerant patients was higher than that in intolerant patients, and the total content of SCFAs is associated with F/B. The production of SCFAs may be related to the higher abundance of genera such as Parabecteroid, which is associated with butyric acid content; genus Oscillospira is associated with overall short-chain fatty acid content [21]^.^

## 5. Limitation

Our study has some limitations. First, this is a single-center observation study, and its conclusion does not fully represent the causal relationship between enteral nutrition tolerance and flora. Second, in our research, it was difficult to estimate the sample size as there are few studies on EN tolerance and microbiota in the ICU population. Considering the exploratory nature of the study and the amount of supporting funds, we adopted a small number of samples (10~15 patients in each group). This may result in not showing statistical differences in some parameters in our study. We only tested the stool on day 1 and day 3. The effect of longer periods remains to be investigated. Therefore, we suggest that further studies to expand the sample size should confirm our result. Finally, other intestinal biomarkers, such as intestinal fatty acid binding protein (IFABP) and citrulline, were not tested synchronously in our study [22]. In our ICU, IFABP and citrulline are not clinically available, and their correlation with GI function is inconsistent in other studies. [22,23]. 

## 6. Conclusions

The gut microbiota composition and fecal SCFA content were significantly different in patients with different enteral nutrition tolerance. The ratio of Firmicutes to Bacteroidetes (F/B) was higher in the intolerance group on the first day of EN, and microbiota diversity was higher in the tolerance group. These parameters can be tools for the early prediction of EN tolerance in critical care patients. However, considering the limited research capacity, further research is needed to confirm our results.

## Figures and Tables

**Table 1:** Characteristics of the patients.

	Tolerance *n* = 14	Intolerance *n* = 10	*p*
Age median (IQR)	62 (50, 68)	61 (52, 65)	0.75
Male *n* (%)	7 (50.00%)	6 (60.00%)	0.62
APACHE II median (IOR)	12 (10, 21)	15 (12, 23)	0.26
SOFA median (IQR)	6.5 (9, 4)	9 (6, 14)	0.09
Weight Kg	48 (45, 68)	50 (40, 82)	0.72
mNutric	5 (3, 6)	5 (4, 6)	0.73
Estimated energy intake (Kcal/day) Median (IOR)	1000 (800, 1500)	1000 (800, 1500)	0.74
EN energy intake on day 3 (Kcal/day) Median (IQR)	950 (700, 1000)	500 (300, 600)	0.02
28-day survival *n* (%)	9 (64.28%)	5 (50.00%)	
Comorbidity			
Diabetes	4 (28.57%)	4 (40.00%)	0.55
Hypertension	6 (42.86%)	5 (50.00%)	0.72
COPD	7 (50.00%)	6 (60.00%)	0.62
Coronary vascular disease	5 (35.71%)	6 (60.00%)	0.23
Days before ICU	5 (3, 14)	4 (2, 10)	0.21
Infection site *n* (%)			
Respiratory	11 (78.57%)	5 (50.00%)	0.14
Blood	2 (14.29%)	1 (10.00%)	0.75
Urine	1 (7.14%)	0 (0%)	0.38
Central nervous system	1 (7.14%)	0 (0%)	0.38
Maximal AGI from day 1 to day 3 *n* (%)	6 (42.86%)	3 (30.00%)	0.52 #
Grade I	2 (14.29%)	1 (10.00%)	
Grade II	4 (28.57%)	2 (20.00%)	
Grade III	8 (57.14%)	6 (60.00%)	
Grade IV	0 (0.00%)	1 (10.00%)	
GI symptoms from day 1 to day 3 *n* (%)			
Nausea/vomiting	5 (35.71%)	6 (60.00%)	0.23
Diarrhea	3 (21.43%)	4 (40.00%)	0.32
Abdominal distension	5 (35.71%)	5 (50.00%)	0.48
Organ support			
Mechanical ventilation	6 (42.86%)	6 (60.00%)	0.40
Vasopressor	10 (71.43%)	8 (80.00%)	0.63
Antibiotic regimen *n* (%)			
Carbapenems	10 (71.42%)	6 (60.00%)	0.56
The 3rd/4th generation of cephalosporin	2 (14.29%)	3 (30.00%)	0.35
β-lactams/β-lactamase inhibitors	2 (14.29%)	1 (10.00%)	0.75
Vancomycin	4 (28.57%)	3 (30.00%)	0.97
Combination of any two above	4 (28.57%)	3 (30.00%)	0.97
Pathogen *n* (%)			
Staphylococcus aureus	1 (7.14%)	1 (10.00%)	0.80
Candida	3 (21.43%)	2 (20.00%)	0.93
Enterobacteria	1 (7.14%)	3 (30.00%)	0.19
Acinetobacter	5 (35.72%)	5 (50.00%)	0.48
Undefined	2 (14.29%)	2 (20.00%)	0.71

AGI, acute gastrointestinal injury; IQR, interquartile range; #, Mantel–Haenszel test.

**Table 2:** The top ten relative abundances of tolerant and intolerant patients on genus and phylum levels.

	Day 1		*p*	Day 3		*p*
	Tolerance	Intolerance		Tolerance	Intolerance	
genus level						
Enterococcus	12.37%	25.59%	0.01 *	15.78%	39.10%	0.01 *
Bacteroides	20.93%	5.59%	0.01 *	13.46%	12.42%	0.8
Escherichia-Shigella	7.86%	2.91%	0.12	13.14%	0.16%	0.01 *
Alistipes	3.21%	1.61%	0.46	6.72%	6.29%	0.20
Klebsiella	0.43%	9.52%	0.01 *	1.53%	5.49%	0.12
Bifidobacterium	4.11%	6.88%	0.39	6.48%	0.62%	0.02 *
Parabacteroides	7.89%	0.32%	0.00 *	4.09%	1.83%	0.33
Sphingomonas	0.13%	2.31%	0.16	0.04%	9.73%	0.01 *
Erysipelatoclostridium	1.36%	0.32%	0.42	6.29%	0.34%	0.02
Subdoligranulum	2.43%	1.90%	0.79	2.18%	1.99%	0.92
Others	39.29%	43.05%	0.58	30.29%	22.03%	0.18
phylum level						
Firmicutes	43.73%	52.75%	0.20	47.97%	57.38%	0.18
Bacteroidetes	33.52%	8.08%	0.00*	25.69%	20.79%	0.41
Proteobacteria	10.54%	24.39%	0.09	15.16%	17.82%	0.61
Actinobacteria	8.25%	14.44%	0.16	7.60%	3.72%	0.23
Verrucomicrobia	2.93%	0.03%	0.08	2.80%	0.01%	0.09
Euryarchaeota	0.58%	0.00%	1.00	0.70%	0.00%	1.00
Synergistetes	0.19%	0.01%	1.00	0.01%	0.00%	1.00
Chloroflexi	0.07%	0.05%	0.55	0.01%	0.07%	1.00
Acidobacteria	0.03%	0.06%	0.21	0.01%	0.05%	0.21
Gemmatimonadetes	0.05%	0.03%	0.72	0.01%	0.01%	1.00
Others	0.11%	0.16%	0.44	0.05%	0.15%	0.04 *

*: *p* < 0.05.

**Table 3:** Firmicutes/Bacteroidetes ratio between tolerant and intolerant patients.

	Firmicutes Abundance	Bacteroidetes Abundance	F/B	*p*
Day 1 Tolerance (a)	26.38 (14.89, 36.17)	11.49 (1.14, 19.92)	1.96 (0.63, 12.6)	a vs. b 0.72
Day 3 Tolerance (b)	20.41 (17.09, 37.69)	3.82 (0.29, 38.33)	5.15 (0.29, 48.26)	b vs. d 0.08
Day 1 Intolerance (c)	37.18 (11.12, 43.98)	0.63 (0.04, 4.72)	50.44 (3.95, 104.26)	a vs. c 0.02
Day 3 Intolerance (d)	27.07 (11.10, 50.37)	3.90 (0.17, 11.75)	9.3 (3.16, 25.19)	c vs. d 0.04

**Table 4:** Fecal SCFAs of tolerant and intolerant patients on day 3.

SCFAs (ug/g)	Healthy Control *n* = 3	Tolerant Group *n* = 14 Median (IQR)	Intolerant Group *n* = 10 Median (IQR)	*p* *
Acetic acid	700.25 (758.10, 564.15)	606.45 (948.10, 334.15)	213.08 (514.27, 203.64)	0.06
Propionic acid	450.94 (500.08, 435.23)	422.94 (181.88, 585.23)	55.62 (31.47, 454.28)	0.04 †
Isobutyric acid	57.84 (30.61, 60.3)	42.84 # (14.61, 131.3)	6.20 # (5.81, 45.04)	0.04 †
Isovaleric acid	74.06 (35.86, 89.73)	62.06 (18.76, 169.81)	4.28 (4.00, 56.33)	0.04 †
Butyric acid	360.38 (220.92, 480.02)	241.38 # (107.62, 474.05)	21.41 # (3.52, 250.16)	0.03 †
Valeric acid	45.92 (12.26, 65.40)	21.92 (7.26, 64.39)	4.08 (1.07, 24.23)	0.04 †
Hexanoic acid	13.38 (1.36, 15.34)	9.43 (3.35, 10.79)	6.25 (1.71, 9.71)	0.09
Total SCFA	1780.13 (865.64, 1929.40)	1560.12 # (865.64, 1929.40)	286.82 # (278.62, 1544.27)	0.03

SCFA: short-chain fatty acid; #:Wilcoxon test; *: Jonckheere–Terpstra trend test; †: *p* < 0.05.

## Data Availability

The datasets used and/or analyzed during the current study are available from the corresponding author ErZhen Chen on reasonable request.
